# The impact of artificial intelligence on students’ metacognition: a systematic review

**DOI:** 10.3389/fpsyg.2026.1947198

**Published:** 2026-10-08

**Authors:** Chunyu Ji, Qi Tan

**Affiliations:** 1 School of Mathematical Sciences, East China Normal University, Shanghai, China; 2 School of Mathematics and Statistics, MOE-LCSM, Hunan Normal University, Changsha, China

**Keywords:** metacognition, artificial intelligence, interactive pattern, Learning-Oriented AI, General-Purpose AI

## Abstract

Given the critical role of metacognition in academic achievement and the rapid integration of Artificial Intelligence (AI) in educational settings, understanding the interplay between the two has become essential. In recent years, a growing number of studies have examined their relationship, creating a pressing need for a systematic review to synthesize the accumulating evidence. This review adhered to the PRISMA guidelines, involving the search and screening of papers from three major databases (Web of Science, Scopus, EBSCOhost). A total of 22 empirical studies were included in the final synthesis. Data extraction and thematic analysis were employed to identify key impacts and underlying interaction patterns. This review distinguishes between Learning-Oriented AI (LAI) and General-Purpose AI (GAI) to organize the evidence. The findings suggest that LAI was more often associated with Guided Interaction, in which AI provides guiding feedback without directly supplying answers, whereas GAI was more commonly associated with Direct Feedback Interaction, in which AI provides answers or explicitly corrects errors. Across both categories, positive findings were reported for several metacognitive outcomes, including regulation, awareness, and shared metacognition. However, some GAI studies also reported that students relied excessively on AI, particularly when they accepted direct answers from AI without sufficient reflection. Overall, the findings highlight the importance of interaction design and teacher guidance in shaping how AI supports students’ metacognitive development.

## 1 Introduction

Since 2022, Artificial Intelligence (AI) has garnered global attention for its ability to generate systematic responses that are rich in information (Zhai, 2022). It has shown significant potential in explaining concepts, solving problems, and enhancing student engagement (e.g., Crompton and Burke, 2024; Kasneci et al., 2023; Urban et al., 2024). However, AI also presents challenges. These include a lack of transparency in its decision-making processes (Bearman et al., 2024), as well as issues of inaccuracy and bias in its responses (Hsu and Ching, 2023). Furthermore, students’ overreliance on AI may reduce higher order thinking engagement (Montenegro-Rueda et al., 2023) and lead to declines in cognitive abilities (Adeshola and Adepoju, 2023).

Concurrently, metacognition, described as “thinking about thinking” (Flavell, 1976, 1979), has been confirmed as a key factor influencing academic achievement (Hattie, 2009; Depaepe et al., 2010). After decades of development, metacognition research stands as a mature field (Stillman and Mevarech, 2010). Substantial evidence demonstrates that metacognition profoundly influences students’ achievement across various domains (e.g., Thomas and McRobbie, 2001; Depaepe et al., 2010; Tanner, 2012). Teaching metacognition is identified as one of the three major instructional recommendations from research on human learning (Bransford et al., 2000).

Recently, AI, with powerful analysis in real time and adaptive feedback capabilities, offers dynamic support for students’ learning. While existing literature reviews have predominantly explored the impact of AI on areas such as student motivation (Badarudin et al., 2025), computational thinking (Weng et al., 2024), critical thinking (Melisa et al., 2025), creativity (Deckker and Sumanasekara, 2025), and self-regulated learning (Sardi et al., 2025), a growing number of researchers are now turning their attention to AI’s impact on metacognition. However, existing reviews have generally focused on broader learning outcomes or related constructs, such as self-regulated learning and higher-order thinking, rather than specifically synthesizing how AI interaction designs relate to students’ metacognitive processes. Therefore, this review focuses on AI-supported metacognitive outcomes and further examines the interaction patterns through which AI may support or constrain students’ metacognitive regulation.

## 2 Background

### 2.1 General-Purpose AI and Learning-Oriented AI

This section aims to differentiate between General-Purpose Artificial Intelligence (GAI) and Learning-Oriented Artificial Intelligence (LAI). LAI refers specifically to artificial intelligence systems that are designed to support student learning. Typically built on technologies such as large language models, LAI systems are infused by their designers with pedagogical principles and domain-specific knowledge to provide instructional support. They can manifest in various forms, including chatbots, learning platforms, or AI-powered software/applications. For instance, the AI chatbot Bubi features a dialogue system that provides question lists and learning diagnostic feedback (Liang et al., 2024); The AI chatbot developed by Yin et al. (2025) was configured with different modes such as metacognitive feedback, affective feedback, and neutral feedback; *Duolingo* is an AI-powered application that helps learners correct their pronunciation and improve grammatical accuracy through instant and accurate voice feedback. In contrast, GAI denotes AI tools with broad applications (e.g., ChatGPT) that are not specifically tailored for educational contexts. They are designed to respond to user queries by providing instant solutions, with the primary objective of generating fluent and coherent responses as efficiently as possible.

We acknowledge that this binary distinction is a heuristic simplification. In practice, a GAI system can be pedagogically configured through prompts or system instructions, potentially making its functional role similar to that of LAI in a given educational context. Rather than treating LAI and GAI as fixed technological categories, we conceptualize them as ideal types along a continuum, classified according to how the system was used and described in each study. This typology serves as an analytical device to organize our synthesis, not as a claim about what AI systems fundamentally are.

### 2.2 The concept of metacognition

The concept of metacognition was first systematically introduced by Flavell (1976). He defined metacognition as: “one’s knowledge concerning one’s own cognitive processes and products or anything related to them… Metacognition refers, among other things, to the active monitoring and consequent regulation and orchestration of these processes” (p. 232). In short, metacognition is the knowledge about and regulation of one’s cognitive activities throughout learning processes (Flavell, 1979; Brown, 1978).

Although various regulatory skills are described in the literature, most frameworks include three core skills: planning, monitoring, and evaluation (e.g., Schraw and Moshman, 1995; Akturk and Sahin, 2011; Desoete and De Craene, 2019). Planning involves selecting goals, choosing appropriate strategies, and allocating resources that influence performance (Schraw and Moshman, 1995; Schraw, 1998). For example, a student may strategically allocate time and attention before starting a task. Monitoring refers to one’s on-line awareness of comprehension and task performance (Schraw and Moshman, 1995; Schraw, 1998). For example, a student may check whether he/she has deviated from the goal or made calculation errors while solving problems. Evaluation refers to appraising the products and efficiency of one’s learning (Schraw, 1998). For example, a student may evaluate the goal attainment and completion after completing a task. Unlike monitoring, the evaluation phase typically occurs after a learning task is completed.

In the present review, the term metacognition serves as a general construct encompassing several related but distinct facets. Following Schraw and Moshman (1995) and Schraw (1998), metacognition broadly includes metacognitive knowledge, metacognitive regulation, and related constructs such as metacognitive awareness. The included studies also operationalized metacognition through more specific outcomes, such as strategy use, sensitivity, reasoning, and socially shared metacognition. Therefore, these outcomes were treated as related but not equivalent indicators of metacognition in the synthesis.

### 2.3 Technology’s impact on students’ metacognition

With technological advances, researchers have increasingly explored how technology affects students’ metacognition. Early studies showed hypermedia environments could enhance metacognition (Azevedo et al., 2008). Mair (2012) found that *Meta-reflection* (anonline tool based on Excel) effectively improved metacognitive skills by combining technological convenience with structured reflection. Wu and Pedersen (2011) demonstrated that computers could scaffold metacognition by structuring tasks and reducing cognitive load, though effectiveness relied on integration with teacher guidance. Similarly, Molin et al. (2020) found *Socrative*, a voting system based on smartphones, required teacher explanations to activate metacognitive monitoring rather than merely displaying answers. Collectively, technological tools partially transformed abstract metacognitive processes into operational steps. However, these technologies primarily functioned as static tools offering fixed procedural guidance without personalized feedback (Zohar and Barzilai, 2013).

Recent advancements in AI have spurred a growing body of studies examining its impact on students’ metacognition. Empirical studies, however, yield mixed results: some indicate that AI can promote students’ metacognition (e.g., Qiao and Zhao, 2023; Azevedo et al., 2022), while others reveal it may inhibit metacognitive development (e.g., Urban et al., 2024; Amani and Bisriyah, 2025). Consequently, there is a critical need to synthesize existing empirical evidence to summarize the impacts of AI on students’ metacognition and analyze the underlying interaction patterns. Therefore, this systematic review aims to address the following questions:

RQ1: What are the positive impacts of AI on students’ metacognition?

RQ2: What are the negative impacts of AI on students’ metacognition?

RQ3: What are the key interaction patterns between students and AI?

## 3 Methods

### 3.1 Identification and screening process

This study employed the Systematic Literature Review (SLR) method, adhering to the Preferred Reporting Items for Systematic Reviews and Meta-Analyses (PRISMA) guidelines. This study was not registered in PROSPERO, which mainly serves health-related reviews, and this aligns with some systematic reviews in education domain (e.g., Hu and Xiao, 2025; Qian et al., 2026). In addition, the completed PRISMA 2020 checklist is provided as Supplementary Material. In accordance with methodological standards recommending the use of three or more databases as sources (Page et al., 2021), searches were conducted in Web of Science, Scopus, and the EBSCOhost platform (see Table 1). The EBSCOhost search was conducted through the institutional platform’s integrated search interface, which simultaneously searched across multiple subscribed databases, including Academic Search Premier, APA PsycINFO, APA PsycArticles, and ERIC. These sources were selected because they provide broad coverage of the interdisciplinary literature relevant to this review, including education, educational psychology, educational technology, and artificial intelligence. The search cutoff date was set to June 6, 2025, with no restrictions on the start date, educational level, or other criteria. The initial search yielded 379 papers (175 from Web of Science, 148 from EBSCO, and 56 from Scopus). After removing 20 duplicates, 359 papers proceeded to the screening stage (see Figure 1).

**Table 1:** Commands used for search.

Database	Search command	Total results
Web of science	TS = (“AI” OR “artificial intelligence” OR “large language models” OR “chatbot” OR “chatGPT”) AND (“metacognition” OR “metacognitive”) AND (“student” OR “education”)	175
EBSCO	(“AI” OR “artificial intelligence” OR “large language models” OR “chatbot” OR “chatGPT”) AND (“metacognition” OR “metacognitive”) AND (“student” OR “education”)	148
Scopus	TITLE-ABS-KEY = (“AI” OR “artificial intelligence” OR “large language models” OR “chatbot” OR “chatGPT”) AND (“metacognition” OR “metacognitive”) AND (“student” OR “education”)	56

**Figure 1:**
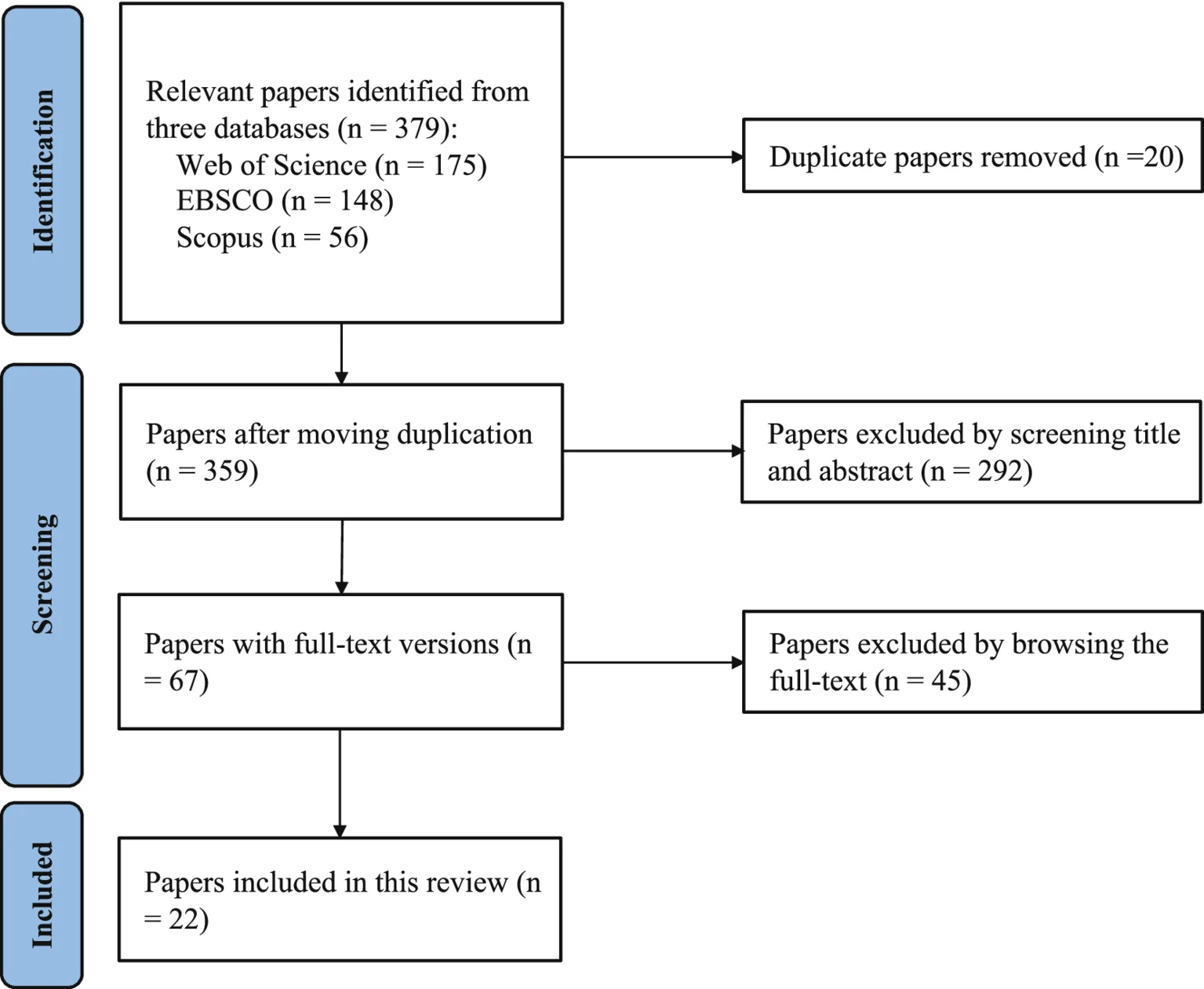
The PRISMA flowchart for the literature search and screening process.

Following the PRISMA protocol, the following inclusion/exclusion criteria were applied during the screening process to ensure that the included literature maintained high academic quality and direct relevance to the research questions:

1. The publication must be a peer-reviewed empirical study; proceedings, conference papers, book chapters, theoretical articles, reviews, and opinion pieces were excluded.
2. The paper must be written in English.
3. The study must explicitly discuss the “impact of AI on student metacognition” in its results or conclusions; studies focusing on the “impact of student metacognition on AI use” were excluded.
4. The paper must explicitly use the term “metacognition” or related phrases (e.g., “metacognitive awareness,” “metacognitive regulation,” “metacognitive strategies”) in its results or conclusions; studies that only implicitly address metacognitive concepts without clear articulation were excluded.
5. Studies published in 2025 were included only if they had undergone peer review and were publicly available before the search cutoff date, including advance online publications. Unpublished preprints were excluded.

The screening process consisted of two rounds: based on the title and abstract of each paper, the first round of screening focused on criteria 1–2, retaining 67 papers. The second round involved examining the full text of these 67 papers, focusing on criteria 3–4. Ultimately, 22 papers were included in this systematic review. Figure 1 presents the PRISMA flowchart detailing the search and screening process. To ensure the reliability of the screening results, two researchers independently conducted the process, achieving an initial agreement rate of over 90%. Any discrepancies were resolved through discussion until consensus was reached.

### 3.2 Data coding and analysis process

During the coding process, basic data were extracted from each included study, including the basic information (including author(s), year, title), the type of AI used in the study (LAI/GAI), the types of metacognitive outcomes, as well as the effects of AI on students’ metacognition. Given the relatively small number of included studies, both authors independently coded all 22 studies rather than coding a subsample, and disagreements during coding were resolved through discussion. Finally, an inductive thematic analysis, following the approach proposed by Braun and Clarke (2006), was employed to derive student-AI interaction patterns from the literature. The process consisted of the following six steps: (1) Familiarising: the researchers thoroughly read the full text of all 22 included papers, with a particular focus on the interaction methods between AI and students. (2) Generating initial codes: segments of text describing interaction processes between AI and students were coded into concise labels, resulting in initial codes (e.g., “AI provides feedback after evaluation”). (3) Searching for themes: related initial codes were grouped together. (4) Reviewing themes: the identified themes were refined and consolidated. (5) Defining and naming themes: each theme was clearly defined and assigned a distinct name. This process ultimately identified two central themes: “Guided Interaction” and “Direct Feedback Interaction”. (6) Writing up: the findings were synthesized into the results reported in this review. The extracted study characteristics are summarized in Supplementary Appendix B.

### 3.3 Methodological quality appraisal

The methodological quality of the included studies was appraised using the Mixed Methods Appraisal Tool (MMAT), as developed by Hong et al. (2018). The MMAT was selected because the review included qualitative studies, randomized controlled trials, quantitative non-randomized studies, quantitative descriptive studies, and mixed-methods studies. Following the MMAT procedure, each study was evaluated against the five criteria corresponding to its methodological design. Each criterion was rated as “Yes,” “No,” or “Cannot tell”. Two reviewers independently classified the study designs and conducted the appraisal. Disagreements were resolved through discussion until consensus was reached. The complete appraisal is presented in Supplementary Appendix A. Overall, the included studies generally demonstrated satisfactory methodological quality, although some methodological limitations were identified across individual criteria. Specifically, 8 studies met all five criteria, 10 met four criteria, 3 met three criteria, and 1 met two criteria (Table 1).

## 4 Findings

Based on a review of 9 papers investigating LAI and 13 examining GAI, this section presents the findings by analyzing three key aspects emerging from student-AI interactions: their positive impacts on metacognition, their negative impacts, and the interactive patterns that underlie them.

### 4.1 Positive findings

As shown in Table 2 and 3, all 9 relevant studies reported findings suggesting potential benefits of LAI for students’ metacognition. However, these findings were derived from diverse research designs and measurement approaches, including experimental comparisons, self-reported measures, qualitative accounts, and observational evidence. Therefore, the reported findings should be interpreted as indicating potential benefits rather than equivalent evidence of causal impact. In addition, the MMAT appraisal indicated that most studies met four or five methodological criteria, suggesting generally satisfactory methodological quality. Nevertheless, some variation existed across studies, and findings from studies with fewer methodological strengths were interpreted more cautiously rather than considered equivalent to those from higher-quality studies. Given the conceptual diversity of metacognition, the following synthesis is organized according to the primary constructs measured in the included studies rather than treating all metacognitive outcomes as interchangeable (Table 3).

**Table 2:** Key findings of LAI.

Author	AI tool	Primary metacognitive construct measured	Positive findings	Negative findings
Qiao and Zhao (2023)	AI-based application *Duolingo*	Metacognitive strategies	The group using *Duolingo* demonstrated significantly higher performance in metacognitive strategies than the control group.	None reported
Liang et al. (2024)	AI-based chatbots “Bubi”	Metacognitive awareness	The group assisted by Bubi showed significantly higher metacognitive awareness than the control group.	None reported
Yin et al. (2025)	AI-driven Chatbot	Metacognitive sensitivity	The group receiving metacognitive feedback from AI showed significantly higher metacognitive sensitivity than the control group.	None reported
Edwards et al. (2025)	An AI designed by author team	metacognitive awareness	AI can enhance group-level metacognitive awareness.	AI’s proactive interventions could cause negative perceptions (e.g., perceived repetitiveness of prompts).
Neshaei et al. (2025)	Intelligent system reflectium	*metacognitive skills*	Compared to the control group, students with AI support showed more significant improvement in both the breadth and depth of reflection.	None reported
Kim J. et al. (2025)	Multiple AIs	metacognitive regulation	AI can effectively enhance collective metacognition at all stages of group collaborative learning.	None reported
Jin et al. (2023)	Multiple AIs	Metacognitive regulation	AI can foster students’ metacognitive development across the planning, performance, and reflection phases.	None reported
Chen (2024b)	AI chatbot Replika	Metacognitive awareness	Compared to the control group, students using AI more effectively enhanced their metacognitive awareness.	None reported
Ortega-Ochoa et al. (2024)	AI chatbot DSLab-Bot	Metacognitive reasoning	The feedback provided by AI was positively correlated with multiple dimensions of student metacognition.	None reported

**Table 3:** Key findings of GAI.

Author	AI tool	Primary metacognitive construct measured	Positive findings	Negative findings
Yao et al. (2025)	ChatGPT	Metacognitive strategies	Using ChatGPT can promote the application of various metacognitive strategies in students’ writing.	None reported
Fan et al. (2025)	ChatGPT	Metacognitive engagement	None reported	The frequency of metacognitive activities among students using AI is lower than that of students using task lists.
Urban et al. (2024)	ChatGPT	Metacognitive monitoring accuracy	None reported	The use of AI leads to a disconnect between students’ self-evaluation and their objective performance.
Dahri et al. (2024)	ChatGPT	Metacognitive self-regulated learning	In stages such as planning, monitoring, and reflection, ChatGPT can provide significant support for students’ metacognitive regulation.	None reported
Iqbal et al. (2025)	Not specified	Shared metacognition	AI can enhance shared metacognition among students.	None reported
Teng (2025)	ChatGPT (via Poe)	Metacognitive awareness	Students using ChatGPT showed significantly higher metacognitive awareness than the control group.	Students with low metacognitive awareness struggle to fully utilize AI capabilities, leading to blind reliance.
Zhan and Yan (2025)	ChatGPT	Metacognitive engagement	None reported	AI feedback can lead to low metacognitive engagement, lack of self-monitoring, and reflection.
Amani and Bisriyah (2025)	ChatGPT/Gemini	Metacognitive strategies	Using AI can promote learners’ metacognitive strategies in writing (including planning, monitoring, and evaluation).	None reported
Kim Y. R. et al. (2025)	ChatGPT	Metacognitive activities	Targeted training can promote students’ critical evaluation, self-monitoring, and self-regulation.	None reported
Hwang et al. (2025)	ChatGPT	Metacognitive tendency	The group that received systematic training scored significantly higher in metacognition than the control group.	None reported
Zhu (2025)	ChatGPT	Metacognitive regulation	Under the guidance provided by instructors, students using ChatGPT can enhance their abilities in metacognitive monitoring, planning, and reflection.	None reported
Chen (2024a)	ChatGPT	Metacognitive awareness	After receiving metalinguistic guidance, ChatGPT can significantly enhance students’ metacognitive awareness.	None reported
Sun and Deng (2025)	ChatGPT	Metacognitive knowledge	Guided by the process provided by teachers, AI can effectively stimulate and enhance students’ metacognitive abilities.	In the field of metacognition, ChatGPT’s outputs may lack depth and adaptability, leading students to worry about their reliability.

#### 4.1.1 Metacognitive regulation

Evidence was strongest for metacognitive regulation, which encompasses learners’ planning, monitoring, evaluation, and strategy adjustment during learning. Among the LAI studies, *Duolingo* and chatbots improved metacognitive strategy use and supported goal setting, progress monitoring, and reflection (Qiao and Zhao, 2023; Jin et al., 2023). The GAI studies similarly showed that ChatGPT could support these regulatory processes, as well as information management, debugging, and task adjustment in writing and broader self-regulated learning contexts (Dahri et al., 2024; Yao et al., 2025; Amani and Bisriyah, 2025; Zhu, 2025). These benefits were most apparent when AI use was structured through prompts designed for specific tasks or through training provided by instructors, rather than being entirely open ended (Kim J. et al., 2025; Hwang et al., 2025; Chen, 2024a).

#### 4.1.2 Metacognitive awareness

A second group of studies focused on students’ awareness of their own metacognitive processes. In the LAI literature, Bubi and Replika increased this awareness (Liang et al., 2024; Chen, 2024b), while a chatbot providing metacognitive feedback improved metacognitive sensitivity, that is, learners’ ability to distinguish correct from incorrect judgments (Yin et al., 2025). In the GAI literature, higher awareness was associated with more effective use of ChatGPT feedback and increased following metalinguistic guidance (Teng, 2025; Chen, 2024a); systematic training also strengthened students’ tendency to engage in metacognitive thinking (Hwang et al., 2025). Taken together, these findings suggest that AI can support learners’ awareness of their own cognitive processes, particularly when feedback or training encourages them to examine and evaluate their thinking more explicitly.

#### 4.1.3 Shared metacognition

At the collective level, AI supported learners’ joint awareness and regulation of group learning. In the LAI studies, multiple AI applications were perceived as supporting socially shared regulation across the forethought, performance, and reflection phases of collaborative learning (Edwards et al., 2025; Kim J. et al., 2025). In the GAI literature, AI use was associated with shared metacognition among preservice teachers, as reflected in collaborative reflection, shared problem-solving, group regulation of tasks, and peer feedback (Iqbal et al., 2025). Although fewer studies examined this construct, the findings suggest that AI can function as a shared regulatory resource by making group goals, progress, and strategy use more visible to collaborators.

### 4.2 Negative findings

As shown in Tables 2 and 3, negative findings were less common and were reported mainly in studies of GAI. These cases are summarized below across three metacognitive dimensions.

#### 4.2.1 Metacognitive regulation

Direct AI assistance may weaken students’ active regulation when it replaces opportunities for self-directed monitoring and reflection rather than prompting these processes. For example, Fan et al. (2025) found that students using ChatGPT engaged less frequently in metacognitive processes, such as orientation and ongoing self-regulation, compared with those using a task list. Similarly, Zhan and Yan (2025) reported that students’ engagement with ChatGPT feedback sometimes involved limited self-monitoring and reflection, which they described as “metacognitive laziness.” In this review, “metacognitive laziness” is used as a descriptive synthesis term referring to reduced engagement in metacognitive regulation, such as limited self-monitoring, reflection, and evaluation when learners rely heavily on AI-generated responses. These findings suggest that readily available AI-generated answers may externalize parts of the regulatory process and reduce opportunities for learners to practise metacognitive strategies. Therefore, these findings should be interpreted as related but distinct manifestations of reduced metacognitive engagement, rather than evidence of a single, uniformly measured construct across studies.

#### 4.2.2 Metacognitive awareness

Students with lower metacognitive awareness relied more on ChatGPT for direct answers and struggled to judge feedback relevance or ask effective questions (Teng, 2025). Limited awareness may thus undermine learners’ ability to evaluate AI output against their own goals. In creative problem solving, ChatGPT improved performance but students’ self- evaluation did not align with actual performance (Urban et al., 2024). Together, these findings suggest that AI assisted gains do not necessarily reflect better metacognitive awareness. Without deliberate checking, learners may miss errors or overestimate their understanding.

#### 4.2.3 Shared metacognition

In group learning, proactive metacognitive prompts were sometimes perceived as repetitive and intrusive, which could reduce learners’ willingness to engage with the agent (Edwards et al., 2025). This finding suggests that AI support for collective metacognition needs to be adaptive to the group’s progress and interaction patterns. Otherwise, externally imposed prompts may interrupt rather than facilitate collaborative regulation.

### 4.3 The interaction patterns between AI and student metacognition

Using thematic analysis of the 22 included studies, we identified two broad interaction patterns: Guided Interaction and Direct Feedback Interaction. Of the 22 studies, 20 provided sufficient information to be classified into one of the five subtypes derived from these two broad patterns, whereas 2 studies did not provide enough interaction detail for classification. To determine the interaction patterns, we examined key features of the AI–student interaction process, including who initiated the interaction, whether AI monitored or evaluated students’ responses, and the form and timing of feedback provided. Studies were assigned based on the overall configuration of these features rather than a single characteristic. Tables 4, 5 summarize how these features were represented across the included studies, particularly the timing of AI responses and whether the feedback was guiding or direct. Of the 20 studies with sufficient information for classification, 5 adopted Guided Interaction and 15 adopted Direct Feedback Interaction.

**Table 4:** Mapping of included studies to LAI interaction features and patterns.

Study	AI monitoring/evaluation timing	AI feedback type	Interaction pattern
Qiao and Zhao (2023)	Real-time monitoring	Direct feedback	Model II(b)
Liang et al. (2024)	Post-input evaluation and response	Guiding feedback	Model I(b)
Yin et al. (2025)	Post-input evaluation and response	Guiding feedback	Model I(a)
Edwards et al. (2025)	Real-time evaluation and monitoring	Guiding feedback	Model I(b)
Neshaei et al. (2025)	Post-input evaluation and response	Guiding feedback	Model I(a)
Kim J. et al. (2025)	Hypothetical real-time monitoring	Guiding feedback	Model I(b)
Jin et al. (2023)	None reported	None reported	None reported
Chen (2024b)	Post-input evaluation and response	Direct feedback	Model II(a)
Ortega-Ochoa et al. (2024)	Post-input evaluation and response	Direct feedback	Model II(a)

**Table 5:** Mapping of included studies to GAI interaction features and patterns.

Study	AI monitoring/evaluation timing	AI feedback type	Interaction pattern
Yao et al. (2025)	Post-input response	Direct answers	Model II(a)
Fan et al. (2025)	Post-input response	Direct answers	Model II(a)
Urban et al. (2024)	Post-input response	Direct answers	Model II(a)
Dahri et al. (2024)	Post-input response	Direct answers	Model II(a)
Iqbal et al. (2025)	None reported	None reported	None reported
Teng (2025)	Post-input response	Direct answers	Model II(a)
Zhan and Yan (2025)	Post-input response	Direct answers	Model II(a)
Amani and Bisriyah (2025)	Post-input response	Direct correction	Model II(a)
Kim Y. R. et al. (2025)	Post-input response	Direct answers	Model II(c)
Hwang et al. (2025)	Post-input response	Direct answers	Model II(c)
Zhu (2025)	Post-input response	Direct answers	Model II(c)
Chen (2024a)	Post-input response	Guiding and direct feedback	Model II(c)
Sun and Deng (2025)	Post-input response	Direct answers	Model II(c)

#### 4.3.1 Guided interaction

In the “Guided Interaction” pattern, AI does not directly provide students with the correct answers or explicitly correct their errors. Instead, it offers guiding feedback. Guided Interaction was identified in 5 studies, including 3 studies classified as Pattern I(a) and 2 as Pattern I(b). As illustrated in Tables 4, 5, both subthemes were observed in studies related to LAI.

Three studies adopted Pattern I(a) (Figure 2). In this pattern, the AI first assigns tasks to students. After completing the tasks, students submit their responses to the AI for evaluation. The AI then provides guided feedback rather than direct answers, thereby supporting students in refining their responses through further reflection and revision. For example, Yin et al.’s (2025) chatbot prompted students to reflect on their understanding by asking, “Are you confident of mastering the above content?” Neshaei et al. (2025) drew on the Gibbs reflective cycle, with the chatbot providing prompts tailored to each stage, such as, “Can you describe what happened?”, to support students’ reflection and revision. Students then engage in metacognitive activities such as reflection, to revise their answers before resubmission, thereby creating an iterative improvement cycle. As cycles increase, AI guidance becomes more detailed: Bubi chatbot progresses from brief reminders on first error to metacognitive feedback highlighting common mistakes by the fourth attempt (Liang et al., 2024).

**Figure 2:**
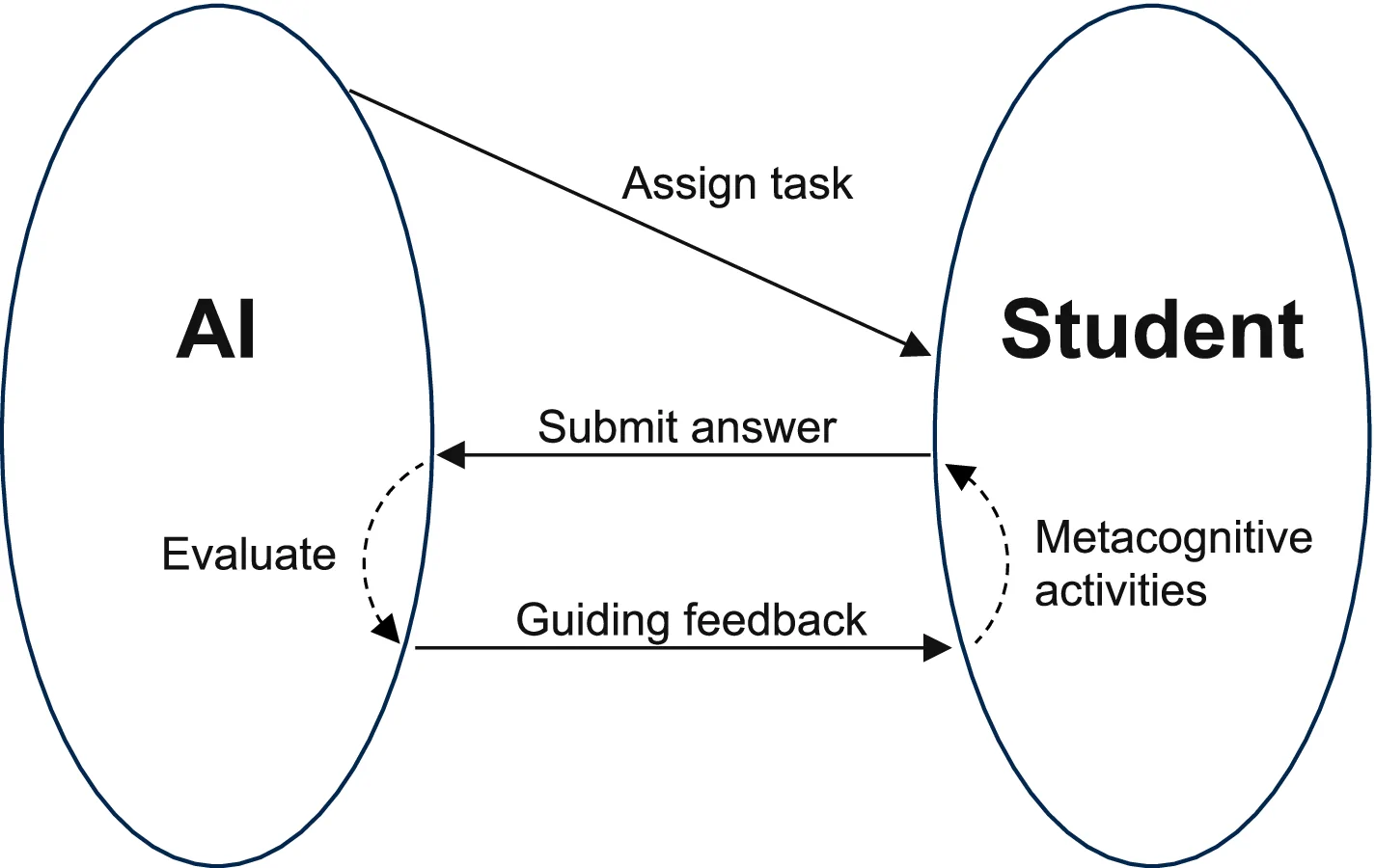
Student-AI metacognitive interaction pattern I(a).

2 studies employed Pattern I(b) (Figure 3). In this pattern, the AI first assigns a task to students and then it monitors students’ responses and provides timely metacognitive feedback. When signs of difficulty or confusion are detected, the AI proactively intervenes with prompts such as, “I notice that you might be confused. Can you explain what are you trying to understand now?” (Edwards et al., 2025). Kim J. et al. (2025) found that LAI monitors students’ learning progress by analyzing behavioral data through learning analytics and interactive dashboards, and provides feedback that supports strategy adjustment.

**Figure 3:**
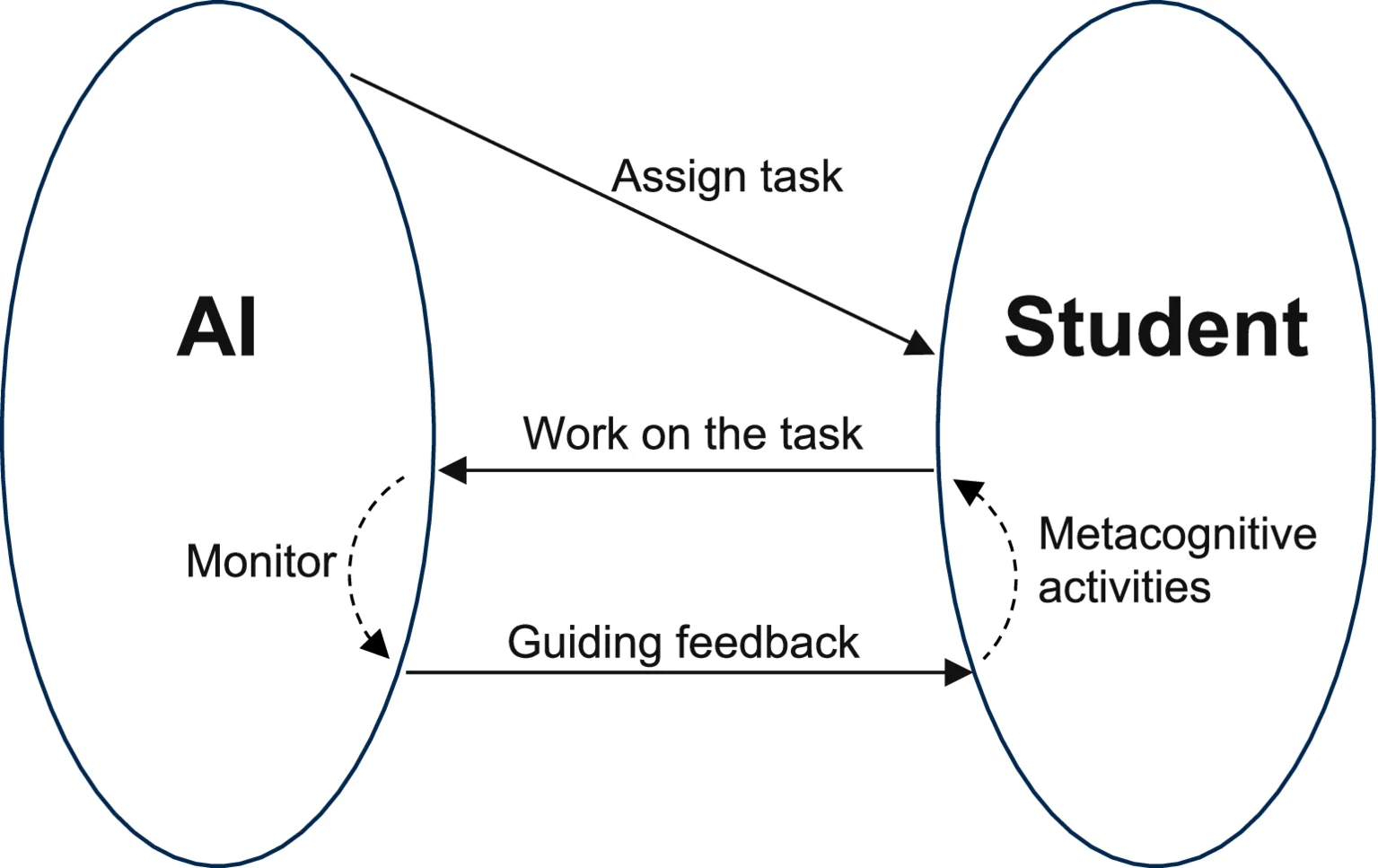
Student-AI metacognitive interaction pattern I(b).

It is noteworthy that all five studies mentioned above reported positive metacognitive outcomes associated with Guided Interaction (see Table 2). These findings suggest that Guided Interaction was frequently associated with students’ metacognitive engagement in the reviewed studies. However, given the limited number and methodological diversity of included studies, this pattern should be interpreted as an observed interaction characteristic rather than evidence that Guided Interaction is inherently superior to other forms of AI support.

#### 4.3.2 Direct feedback interaction

In the “Direct Feedback Interaction” pattern, AI directly provides answers or explicitly corrects students’ errors. This pattern was identified in 15 studies, including 9 studies classified as Pattern II(a), 1 as Pattern II(b), and 5 as Pattern II(c).

As indicated in Table 4, a total of 9 papers adopted Pattern II(a) (Figure 4). Within this pattern, students take the initiative to ask the AI questions in order to obtain conceptual explanations, generate new ideas, acquire suggestions for solving problems, etc. (Fan et al., 2025; Urban et al., 2024; Amani and Bisriyah, 2025). The AI analyzes the queries and provides direct feedback to the students. After engaging in metacognitive activities such as monitoring and evaluating the responses, the students then decide whether to continue asking further questions to the AI.

**Figure 4:**
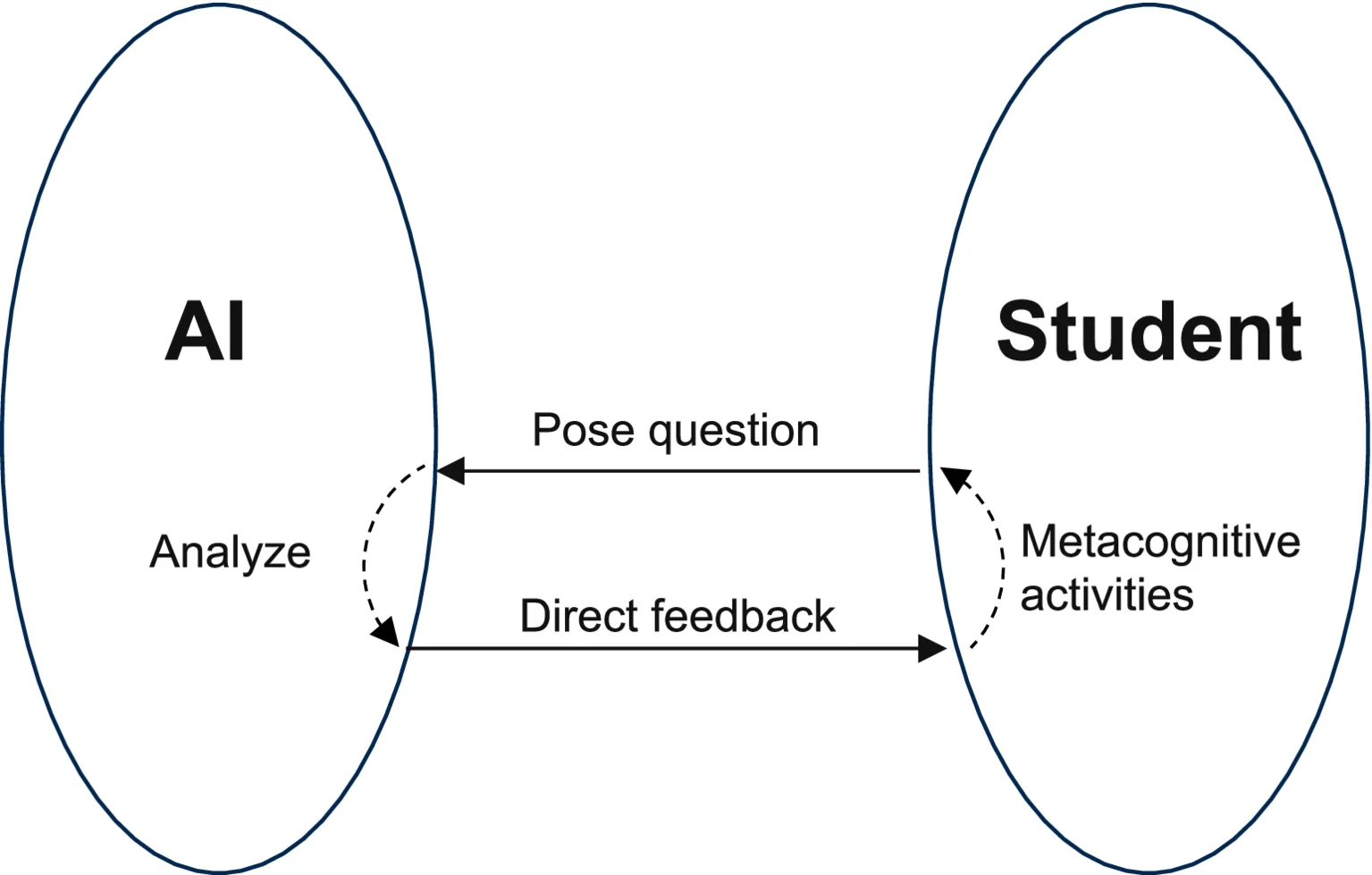
Student-AI metacognitive interaction pattern II(a).

Only one paper adopted Pattern II(b) (Figure 5). In this pattern, the AI assigns tasks to students, who then submit their responses to the AI. The AI immediately evaluates the students’ performance across multiple dimensions such as pronunciation, fluency, vocabulary, and grammatical accuracy (Qiao and Zhao, 2023). Then, the AI provides direct feedback, such as displaying correct answers and explicitly correcting errors, without involving metacognitive feedback. As a result, this pattern does not form an iterative loop.

**Figure 5:**
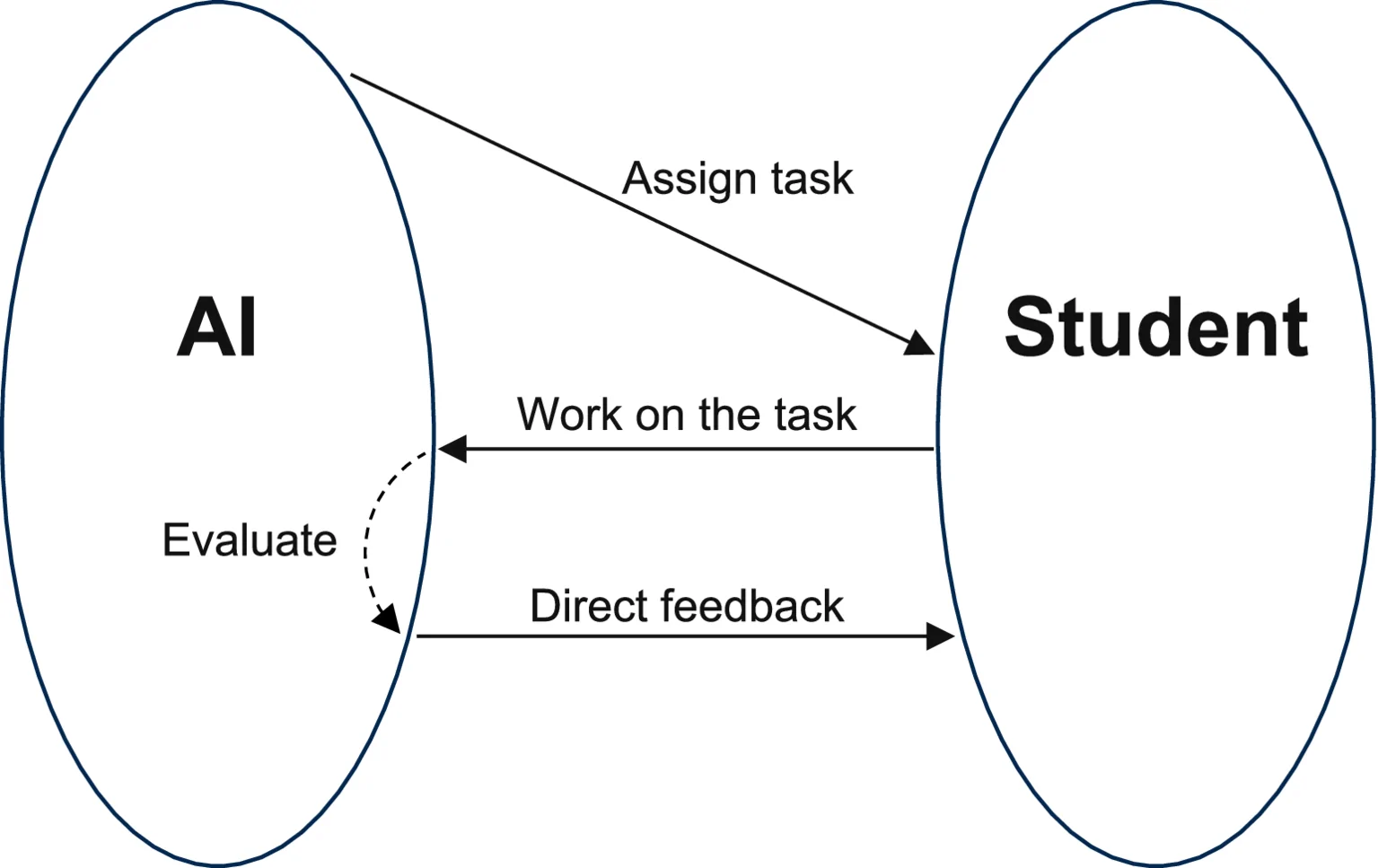
Student-AI metacognitive interaction pattern II(b).

Five papers adopted Pattern II(c) (Figure 6). Unlike Pattern II(a), its key distinction lies in students receiving educator training before AI interaction. In both patterns, to pose effective questions, students must reflect: “What don’t I understand? What help do I need? How to phrase for AI comprehension? Does the response address my confusion?” This self-questioning itself constitutes metacognitive activity. When AI responses fall short, students revise prompts and ask again (Sun and Deng, 2025), making this iterative process valuable for metacognitive development. These activities rely on students’ foundation of metacognitive awareness, established through pre-training in Pattern II(c), such as Hwang et al.’s (2025) approach with four stages encompassing “clarity, context, specificity, and attention balance”.

**Figure 6:**
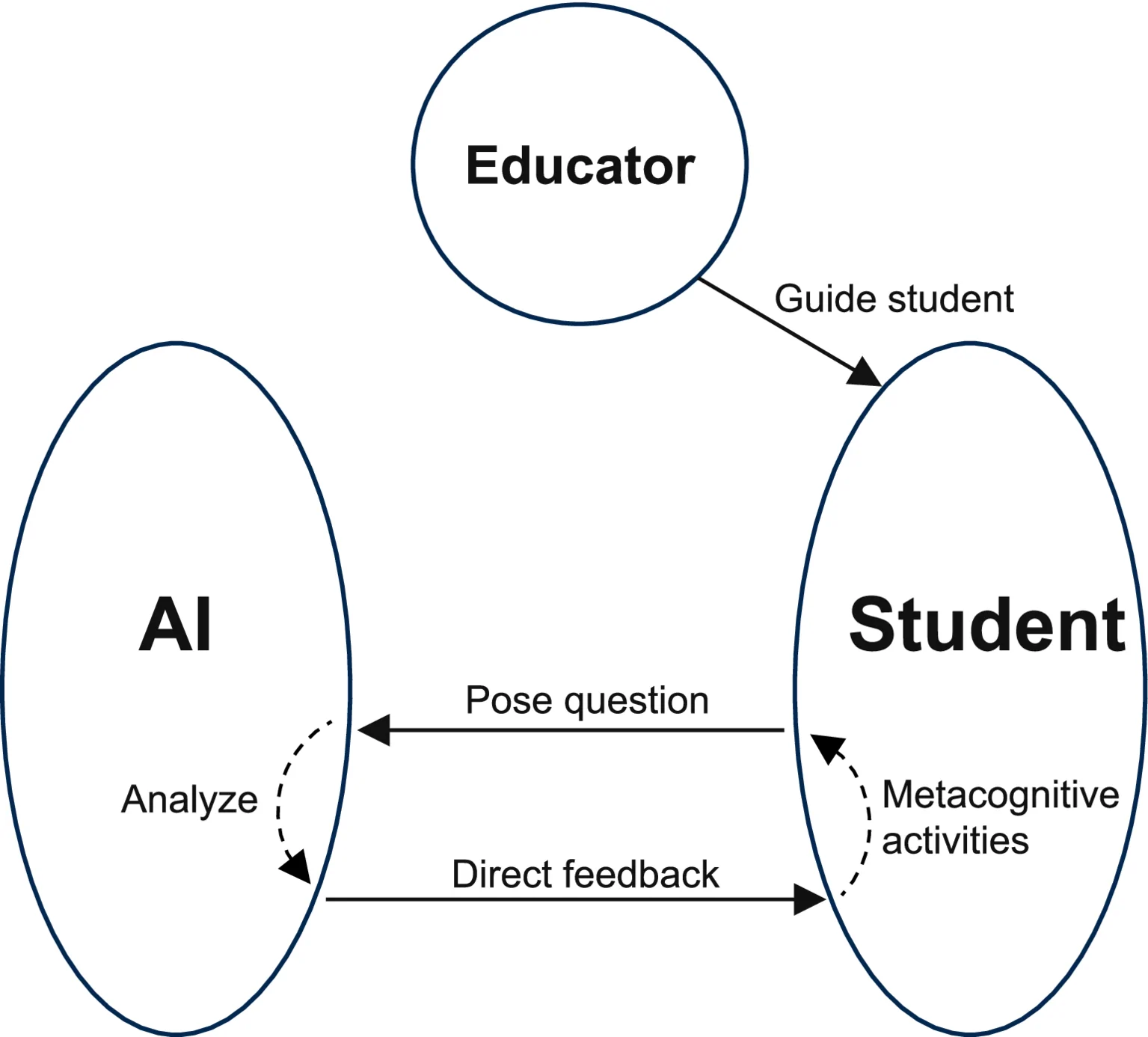
Student-AI metacognitive interaction pattern II(c).

Within the studies included in this review, 12 studies on GAI employed the “Direct Feedback Interaction” pattern, as did one study on LAI. Unlike “Guided Interaction,” AI in this pattern primarily provides direct responses rather than guiding students through a sequence of questions. However, the metacognitive value of this interaction pattern may depend on whether students actively evaluate, question, and reflect on AI-generated responses. In some studies, limited follow-up interaction was observed, suggesting that direct feedback may provide fewer opportunities for metacognitive engagement when students rely on AI outputs without further reflection.

## 5 Discussion

The reviewed studies suggest different patterns in how LAI and GAI were configured to support students’ metacognition. LAI studies more often incorporated structured metacognitive scaffolding and guided interaction, whereas GAI studies more often involved direct feedback and student-initiated questioning. However, the distinction between these interaction patterns should not be interpreted as a distinction between beneficial and detrimental approaches. Rather, the metacognitive effects of AI depend on how feedback is designed, how learners engage with AI responses, and whether sufficient opportunities for self-monitoring and reflection are preserved.

However, although most studies demonstrated satisfactory methodological quality, the MMAT appraisal revealed some variation across individual studies, indicating that the strength of evidence supporting these patterns was not uniform. This observation aligns with the findings of Bastani et al. (2025): AI with a standard interface (like ChatGPT) can significantly impede learning outcomes, while AI Tutors (providing hints rather than direct solutions) help mitigate this risk. Schraw (1998) recommends using the “Regulatory Checklists” as scaffolding tools to guide students in self-questioning and self-regulation. Conceptually, LAI resembles a “Regulatory Checklist powered by AI”, with its core design principle centered on guiding students to complete metacognition by following its scaffold. Furthermore, both LAI and GAI leverage powerful analytical capabilities to provide personalized guidance. This represents a key distinction between AI and earlier technologies (e.g., hypermedia environments, Azevedo et al., 2008), which cannot dynamically infer context or generate adaptive feedback based on user input.

The core mechanism linking AI interaction design to metacognition lies in how the design regulates cognitive offloading. Guided prompts, characteristic of LAI, serve as scaffolding that externalizes metacognitive regulation into actionable steps, including planning, monitoring, and evaluation (Schraw and Moshman, 1995), thereby enabling deliberate practice of self-regulated learning (Zimmerman, 2002). This structured support distributes cognitive load without removing desirable difficulties, fostering strategy internalization. In contrast, direct answers from GAI invite cognitive offloading (Risko and Gilbert, 2016), shifting the burden of verification and reflection to the AI, thus reducing opportunities for active monitoring and evaluation. At the collaborative level, guided interaction functions as a mediating tool for socially shared regulation, making group processes transparent, while direct feedback offers no such mediation. Hence, the decisive distinction is whether AI makes the process of thinking visible or merely supplies the product of thought.

Compared to traditional teacher-student interaction, the student-AI interaction pattern can alleviate students’ fear of asking questions (Steinert et al., 2024), allowing them to seek feedback more freely (Tai and Chen, 2024). However, it also presents certain drawbacks: collaboration between teachers, learners, and technology is crucial (Riedl, 2019), but in this model, the teacher is excluded from the interaction loop, hindering timely teacher supervision and the evaluation of student performance. Based on these observations, we offer a conceptual proposition that teachers might be appropriately integrated into the student-AI interaction to form a metacognitive dynamic involving students, teachers, LAI, and GAI. In this proposed framework, students could choose to interact with either the teacher or the LAI/GAI based on their specific needs. It should be emphasized that this model is a conceptual implication derived from the synthesis of the reviewed studies, rather than an empirically validated framework.

Furthermore, Self-Regulated Learning (SRL) is a concept closely intertwined with metacognition. SRL refers to the process by which learners utilize their mental abilities to transform them into academic skills through self-directed goals (Zimmerman, 2002). Empirical studies have demonstrated AI’s potential to support students in developing SRL (e.g., Lan and Zhou, 2025; Ng et al., 2024; Sardi et al., 2025). However, while SRL provides an actionable framework, metacognition, which serves as its core mechanism, is more implicit and challenging to observe (Desoete, 2008). It becomes internalized gradually as students experience cognitive conflicts and engage in reflection of “what I know and don’t know” and “how to know”. It is this fundamental distinction that leads to the uniquely complex impact of AI on students’ metacognition revealed in this study.

## 6 Conclusion and limitations

Based on a systematic review of 22 empirical studies, this study thoroughly investigates the impact of AI on students’ metacognition. The main conclusions can be summarized as follows:

First, positive findings were reported across multiple dimensions of metacognition, including regulation, awareness, and shared metacognition. LAI more often supported these processes through structured scaffolding and guiding feedback, whereas GAI was also associated with positive metacognitive outcomes when its use was structured through specific prompts, training, or instructor support.

Second, several studies identified potential risks to students’ metacognition. Direct AI assistance may reduce students’ engagement in self-monitoring, reflection, and evaluation, while limited metacognitive awareness may lead to overreliance on AI or inaccurate self-evaluation.

Third, this study identified two types of student-AI interaction patterns: “Guided Interaction” and “Direct Feedback Interaction”. The distinction between the two patterns lies primarily in the role of AI feedback during the interaction, rather than whether metacognitive processes occurred after AI support. Within the reviewed studies, LAI mostly adopts “Guided Interaction,” while GAI mostly employs “Direct Feedback Interaction”. Through proactive questioning, the “Guided Interaction” pattern externalizes abstract metacognition into actionable steps to stimulate students’ metacognitive activities. In “Direct Feedback Interaction,” students lead the questioning, and the AI provides direct, immediate feedback. Throughout this process, students engage in spontaneous metacognitive activities such as monitoring and evaluation.

This study proposes a conceptual typology of LAI and GAI to organize the different ways in which AI may relate to students’ metacognition. Practically, it offers guidance for key stakeholders: For students, they should develop the ability to critically evaluate content generated by AI, rather than passively accepting AI suggestions. For teachers, they need to actively integrate into student-AI interactions by providing timely guidance. LAI designers should address limitations such as repetitive, predesigned prompts and narrow course-specific applications, which can reduce engagement and hinder skill transfer, a core aspect of metacognition.

On future research directions, two key areas warrant attention. First, most included studies focused on immediate or short-term outcomes, and information regarding follow-up periods and sustained effects was rarely reported. Although intervention duration varied across studies (see Supplementary Appendix B), the available evidence remains insufficient to determine whether AI-supported metacognitive changes can be maintained over longer periods. Future longitudinal research is therefore needed to examine whether students can internalize AI-supported metacognitive strategies and maintain self-regulatory habits after AI support is withdrawn. Second, greater attention should be paid to contextual and group differences, as AI’s effects are likely to vary significantly. Future studies should explore these variations in depth by examining, for instance, how metacognitive scaffolding through LAI differs between subjects requiring logical reasoning (e.g., mathematics) and those emphasizing creative expression (e.g., language).

This review has some limitations. First, the inclusion criterion required explicit use of the term ‘metacognition’ or its derivatives in the results or conclusions. While this criterion was adopted to ensure conceptual clarity, it may have excluded studies investigating metacognitively relevant processes such as self-monitoring, self-regulation, reflection, or evaluative judgement without using the specific term. Consequently, the review may not fully capture the broader empirical work on AI and metacognitive processes. Future reviews could broaden the search strategy to include these related constructs. Second, differences in terminology used to describe AI-based educational technologies may have resulted in the omission of some relevant studies, as not all studies may have used the AI-related terms included in the search strategy. Third, restricting the review to peer-reviewed studies published in English may have introduced publication and language bias, meaning that the observed LAI/GAI patterns may not fully represent broader global trends. Fourth, although this study focuses on the latest advances of AI, the pace of AI development far exceeds the academic publishing cycle (Atherton et al., 2024). Both LAI and GAI technologies may undergo “significant advances” within a short time, which may further limit the long-term applicability of the conclusions presented herein. This trend is also reflected in the publication years of the included studies, as 19 of the 22 included studies were published in 2024 and 2025, highlighting the rapidly evolving nature of this research field.

Overall, this review suggests that the impact of AI on students’ metacognition depends not only on the technology itself, but also on how it is used. This further highlights the important role of teachers in guiding students to use AI appropriately to better support their metacognitive development.
