# Supplementary material for: The impact of artificial intelligence on students’ metacognition: a systematic review
Source: Front Psychol. 2026 Oct 8;17:1947198. doi: 10.3389/fpsyg.2026.1947198 (PMC13687014; doi:10.3389/fpsyg.2026.1947198)
Supplement: Supplementary file 1 [file Data_Sheet_1.docx]

**Appendix A. Methodological quality appraisal using the MMAT**

| **Study design** | **Authors (year)** | **C1** | **C2** | **C3** | **C4** | **C5** | **Yes count** |
| --- | --- | --- | --- | --- | --- | --- | --- |
| Qualitative | Kim, Detrick, et al. (2025) | Y | Y | Y | Y | Y | 5 |
| Qualitative | Jin et al. (2023) | Y | Y | Y | Y | Y | 5 |
| Qualitative | Zhu (2025) | Y | Y | Y | Y | Y | 5 |
| Qualitative | Yao et al. (2025) | Y | Y | Y | Y | Y | 5 |
| Qualitative | Zhan & Yan (2025) | Y | Y | Y | Y | Y | 5 |
| Qualitative | Amani & Bisriyah (2025) | Y | Y | Y | Y | CT | 4 |
| Randomized controlled trial | Qiao & Zhao (2023) | Y | Y | Y | CT | Y | 4 |
| Randomized controlled trial | Yin et al. (2025) | Y | CT | Y | CT | Y | 3 |
| Randomized controlled trial | Neshaei et al. (2025) | Y | Y | Y | CT | Y | 4 |
| Randomized controlled trial | Fan et al. (2025) | CT | Y | Y | N | N | 2 |
| Randomized controlled trial | Urban et al. (2024) | Y | Y | Y | Y | Y | 5 |
| Quantitative non-randomized | Liang et al. (2024) | N | Y | Y | N | Y | 3 |
| Quantitative non-randomized | Chen (2024b) | N | Y | Y | Y | Y | 4 |
| Quantitative non-randomized | Ortega-Ochoa et al. (2024) | Y | Y | CT | N | Y | 3 |
| Quantitative non-randomized | Chen (2024a) | N | Y | Y | Y | Y | 4 |
| Quantitative descriptive | Iqbal et al. (2025) | Y | N | Y | Y | Y | 4 |
| Mixed methods | Edwards et al. (2025) | Y | Y | Y | Y | CT | 4 |
| Mixed methods | Kim, Park, & Joung (2025) | Y | Y | Y | Y | Y | 5 |
| Mixed methods | Hwang et al. (2025) | Y | Y | Y | CT | Y | 4 |
| Mixed methods | Sun & Deng (2025) | Y | Y | Y | Y | N | 4 |
| Mixed methods | Dahri et al. (2024) | Y | Y | Y | Y | CT | 4 |
| Mixed methods | Teng (2025) | Y | Y | Y | Y | Y | 5 |

*Note.* Y = Yes; N = No; CT = Can’t tell. C1–C5 refer to the five design-specific MMAT questions listed below the table.

**Screening questions**

**S1** Are there clear research questions?

**S2** Do the collected data allow the research questions to be addressed?

**1. Qualitative studies**

**1.1** Is the qualitative approach appropriate to answer the research question?

**1.2** Are the qualitative data collection methods adequate to address the research question?

**1.3** Are the findings adequately derived from the data?

**1.4** Is the interpretation of results sufficiently substantiated by data?

**1.5** Is there coherence between qualitative data sources, collection, analysis and interpretation?

**2. Randomized controlled trials**

**2.1** Is randomization appropriately performed?

**2.2** Are the groups comparable at baseline?

**2.3** Are there complete outcome data?

**2.4** Are outcome assessors blinded to the intervention provided?

**2.5** Did the participants adhere to the assigned intervention?

**3. Quantitative non-randomized studies**

**3.1** Are the participants representative of the target population?

**3.2** Are measurements appropriate regarding both the outcome and intervention (or exposure)?

**3.3** Are there complete outcome data?

**3.4** Are the confounders accounted for in the design and analysis?

**3.5** During the study period, is the intervention administered (or exposure occurring) as intended?

**4. Quantitative descriptive studies**

**4.1** Is the sampling strategy relevant to address the research question?

**4.2** Is the sample representative of the target population?

**4.3** Are the measurements appropriate?

**4.4** Is the risk of nonresponse bias low?

**4.5** Is the statistical analysis appropriate to answer the research question?

**5. Mixed-methods studies**

**5.1** Is there an adequate rationale for using a mixed-methods design to address the research question?

**5.2** Are the different components of the study effectively integrated to answer the research question?

**5.3** Are the outputs of the integration of qualitative and quantitative components adequately interpreted?

**5.4** Are divergences and inconsistencies between quantitative and qualitative results adequately addressed?

**5.5** Do the different components of the study adhere to the quality criteria of each tradition of the methods involved?

**Appendix B Characteristics of Included Studies**

| **Study** | **Educational stage** | **Sample size** | **Discipline/field** | **Intervention/activity duration** |
| --- | --- | --- | --- | --- |
| Qiao & Zhao (2023) | Postsecondary/adult EFL learners | N = 93 | EFL speaking | 13 weeks (weekly) |
| Liang et al. (2024) | Grade 6 students | N = 39 | museum learning | 60-min |
| Yin et al. (2025) | Undergraduate | N = 87 | Biology | One lab session (15 modules) |
| Edwards et al. (2025) | Higher education; preservice teachers | N = 52 | physics | 60 min |
| Neshaei et al. (2025) | Adults with a health/welfare degree | N = 100 | health and welfare | Single online session |
| Kim, Detrick, et al. (2025) | Undergraduate and graduate | N = 30 | online collaborative learning | One focus-group session |
| Jin et al. (2023) | University students | N = 16 | Cross-disciplinary online learning | One interview; mean 45.3 min |
| Chen (2024b) | Undergraduate | N = 60 | EFL semantics | 8 weeks |
| Ortega-Ochoa et al. (2024) | Online higher education | N = 196 | engineering | 14 weeks |
| Yao et al. (2025) | Postgraduate and master | N = 13 | English academic writing | 13-week course |
| Fan et al. (2025) | Undergraduate and graduate | N = 117 | English reading and writing | One lab session |
| Urban et al. (2024) | University students, bachelor and master | N = 145 | creative problem-solving | Single session; mean 26 min |
| Dahri et al. (2024) | Preservice teachers | N = 300 | Teacher education | Single scenario-based task |
| Iqbal et al. (2025) | Bachelor, master, and doctor | N = 465 | Preservice teacher education | Two survey waves, 2 weeks apart |
| Teng (2025) | Undergraduate | N = 40 | EFL writing | One-semester writing course |
| Zhan & Yan (2025) | Undergraduate | N = 16 | IELTS/EFL writing | 40-min writing; 23–77-min revision |
| Amani & Bisriyah (2025) | undergraduate | N = 40 | EFL academic | 30 min interviews |
| Kim, Park, & Joung (2025) | Undergraduate; preservice teachers | N = 42 | teacher education | One homework activity in a 16-week course |
| Hwang et al. (2025) | Undergraduate | N = 56 | Geriatric nursing | 3 weeks |
| Zhu (2025) | Online graduate | N = 17 | Learning design | 15-week course |
| Chen (2024a) | Undergraduate | N = 42 | EFL linguistics | 6 weeks |
| Sun & Deng (2025) | Undergraduate | N = 70 | Business | Single two-prompt ChatGPT activity |

*Note.* N = total study sample.
